# Use of business model potential in Dutch academic medical centres—A case study

**DOI:** 10.1371/journal.pone.0297966

**Published:** 2024-03-15

**Authors:** Ester M. M. Cardinaal, Joey Truijens, Patrick P. T. Jeurissen, Hubert Berden

**Affiliations:** 1 Operating Rooms, Anesthesiology, Pain and Palliative Medicine, Radboud Universitair Medisch Centrum, Nijmegen, The Netherlands; 2 Radboud Universitair Medisch Centrum, Nijmegen, The Netherlands; 3 Radboud Institute of Health Sciences (RIHS), Nijmegen, The Netherlands; Sunway University, MALAYSIA

## Abstract

Academic Medical Centres (AMCs) are large organisations with a complex structure due to various intertwined missions and (public) roles that can be conflicting. This complexity makes it difficult to adapt to changing circumstances. The literature points to the use of business models to address such challenges. A business model describes the resources, processes, and cost assumptions that an organisation makes in order to the delivery of a unique value proposition to a customer/patient. Do AMC business operations managers actually use business models to address challenges and operate in a way that enables AMCs to adapt to changing circumstances? This study explored whether the use of a business model is a starting point for bringing about change in AMC operations. A case study design was considered appropriate to explore the knowledge and experience of business models among business operations managers of Dutch AMCs. Through purposive sampling, participants were invited to participate in a questionnaire to provide in-depth and detailed information about the use of business models in AMCs. Our research showed that a business model can support the complex organisation of an AMC, but the design and use of business models varies. In general, respondents attribute more potential to the use of a business model than they experience in daily practice. The majority consider a business model to be suitable for bringing about change, but see it only sparingly used in their own AMC. This is the first study to provide some initial insights into the use of business models in Dutch AMCs. We can assume that improvements are possible in order to optimise the change potential of business models in AMCs worldwide. In order to successfully implement an innovative business model, the interpretation of the concept of a business model and the creation of a framework of preconditions should be taken into account. Healthcare providers, policy makers or researchers should explicitly identify the environment in which the model will operate. In particular, by identifying the level of readiness for change readiness at all levels of the organisation.

## 1. Introduction

Academic medical centres (AMCs) are large hospital organisations that combine highly complex patient care, biomedical research, and training and education [1]. An international comparison showed that European AMCs face significant challenges, including staff shortages and ongoing internal tensions regarding the allocation of financial resources between patient care, research and education [2]. The literature increasingly points to the added value of business models to address such challenges [3]. A business model describes the resources, processes and cost assumptions that an organisation makes to deliver a unique value proposition to a customer/patient [4]. From this perspective, changing one or more components of a business model can potentially lead to change. However, the complexity of AMCs makes it difficult for them to adapt to changing circumstances. Wietecha et al. conclude that the governance of AMCs is complicated by the simultaneously of multiple business models: “*The AMC is not a ‘three-legged stool’ of patient care*, *research and teaching–a metaphor implying greater similarity of purpose functioning and financing than is the case*. *The ‘legs’ of that stool are distinct and all different business models”*. Wietecha et al. state that AMCs can only be successful if they use several multiple business models simultaneously [5]. However, little is generally known about the use of business models by business operation managers of AMCs [6]. More specifically, little is known about the use of business models in AMCs to address contemporary challenges.

Our study seeks to explore whether the concept of a business model is recognized, valued, used and applied as such by AMC business operations managers. And, whether a business model is used as a tool to initiate change.

## 2. Theoretical background

### 2.1 Dutch academic medical centres

The Netherlands has developed different hospital services for its 17,6 million inhabitants: 1) 7 academic medical centres, 2) 26 top clinical hospitals and, 3) 73 general hospitals. AMCs are large hospitals that provide a significant amount of highly specialised care and have a leading position in tertiary patient care, biomedical scientific research, knowledge development and innovation. The seven Dutch AMCs employ more than 80,000 people, have a combined annual turnover of more than 10 billion euros and treat about 1.2 million patients a year. Top clinical hospitals provide primary care but as well as care that requires specific specialised facilities. Most top clinical hospitals work in collaboration with other hospitals. They also provide training for medical specialists and are often involved in scientific research. General hospitals are regional hospitals that provide mainly primary care and are relatively small, so they do not usually have specialist teams for many types of illness. Around these hospital groups, the landscape also includes outpatient clinics, specialist hospitals, and independent treatment centres. AMCs differ from other Dutch hospitals in a number of ways. Firstly, AMCs are expected to provide a certain level of basic care that supports the educational objectives. The extent to which this is done varies and also depends on the regional context. Second, the large amount of complex tertiary patient care. Thirdly, the AMCs have been entrusted with public tasks as defined in the Higher Education and Scientific Research Act (“Wet op het hoger Onderwijs en wetenschappelijk Onderzoek”). The AMCs receive specific funding for the performance of these public tasks. The AMCs also receive funding for continuing medical education and hospital training courses.

### 2.2 Business models

In 1957, Bellman et. al introduced a business model to represent reality in a model [7]. Da Silva et. al outline the historical perspective of business model development. They note that the term was not widely used for decades. It was not until the 1990s that there was a renewed interest in business models. With the advent of the Internet, there was a need to organise business differently. The use of bespoke business models was seen as a means of shaping new ways of running Internet businesses [8]. According to Wirtz et al, a business model is a simplified and aggregated representation of the relevant activities of a company [9]. Wirtz identifies a number of components relevant to a business model, including strategy, resources, network relationships, customers, value proposition, revenues and value-creating activities. We used these components as the basis for the questionnaire.

### 2.3 Business model innovation in healthcare

Nowadays, the term business model is regularly used in the academic literature, often in combination with innovation or disruptive innovation. Business model innovation is seen as the need to arrive at a new value proposition in response to changing circumstances [3]. Business model innovation is critical to a firm’s ability to achieve growth and long-term viability [10]. Research shows that financially successful companies value business model innovation twice as much as less successful companies, indicating the potential benefits of explicitly using new business models to anticipate change [9]. To understand the concept of business models in the hospital sector, a literature review was conducted by Lopes et al. [11]. They state that a business model: “*helps to describe*, *analyse*, *manage*, *and communicate*: *(i) the value proposition of the hospital for its patients and the other stakeholders; (ii) the ways in which the organisation creates and delivers this value; and (iii) the economic value required to maintain or to regenerate the environmental*, *technical*, *and legal capital*, *together with the strategies of its organisational boundaries*”. The dynamic aspects, the high degree of regulation and the large number of actors in health care are seen as complicating factors for business model innovation. However, empirical research shows that innovation in healthcare can be successfully achieved through the application of business models. For example, a study of healthcare innovation in Indian (teaching) hospitals found that one hospital specialising in cataract surgery had developed a business model whereby paying patients generated enough cash flow to offer free surgery to less well-off patients [12, 13].

## 3. Methodology

The aim of this study was to increase the current knowledge about the use of business models in Dutch AMCs. Wirtz et al. defined a business model as a simplified and aggregated representation of the relevant activities of a company [9]. They defined a number of components that, in their view, characterise a business model. These include strategy, resources, network relationships, customers, value proposition, revenues and value-creating activities. We used these elements as a framework for our questionnaire. The initial questionnaire was developed by two authors (Ester Cardinaal and Joey Truijens). This questionnaire was piloted with two AMC business operations experts. Based on their feedback minor adjustments were made to the wording of the questions (see S1 Questionnaire). Finally, the questionnaire was reviewed by two expert authors (Hubert Berden and Patrick Jeurissen). It was considered appropriate to use a questionnaire to ask business operations managers of all AMCs in the Netherlands about their knowledge and experience with business models. This is partly because it is an exploratory study on a topic on which little research has been done, and partly because business operations managers work across departments to align teams, set goals, implement initiatives and improve processes—helping the organisation to run efficiently and effectively.

The Research Ethics Committee of Radboud University Nijmegen Medical Centre, the Netherlands, confirmed that the above study was conducted out in accordance with the applicable legislation regarding review by an accredited research ethics committee such as the Medical Research involving Human Subjects Act and the Medical Treatment Contracts Act (file number 2022–15824). The Research Ethics Committee of the Medical Center of Radboud University Nijmegen approved the study.

### 3.1 Data collection

To the best of the researchers’ knowledge, little research has been done on this topic. This study is one of the first explorations in this area. It was not intended to include all perspectives (stakeholders) in the study. Therefore, a small research population was chosen. The data was collected using purposive sampling [14]. At least one respondent was included from each AMC. To be included, the respondent had to hold a key position with oversight, experience and executive responsibility for business management operations. We chose this sample because we believe that business operations managers can provide reliable information about the use of business models [15]. The researchers agree that the exploratory research objective has been met now that at least one or more respondents from each Dutch AMC have been included in the study. A total of 31 respondents were invited, of whom 24 completed the questionnaire (See S1 File). To ensure that all participants had the same level of knowledge about business models, background information was provided prior to the questionnaire (See S1 Questionnaire).

Informed consent was incorporated into the digital questionnaire. The first question of the questionnaire concerned the consent statement (see S1 Questionnaire). The consent form was digitally processed and recorded. A Limesurvey questionnaire (online) was administered. Data were collected between June 2021 and June 2022.

### 3.2 Analysis

**Fig 1:**
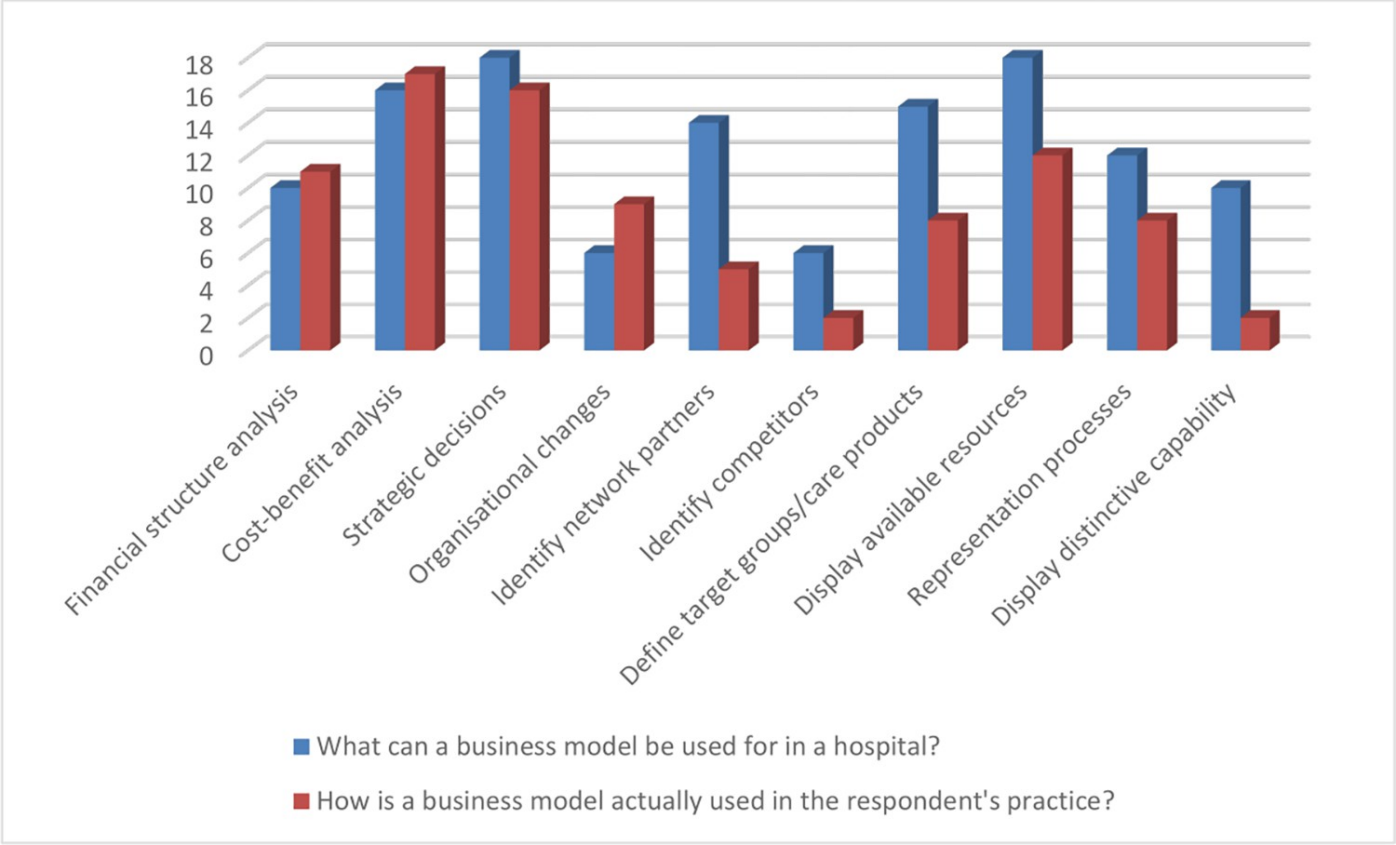
A business model. Use of a business model.

**Fig 2:**
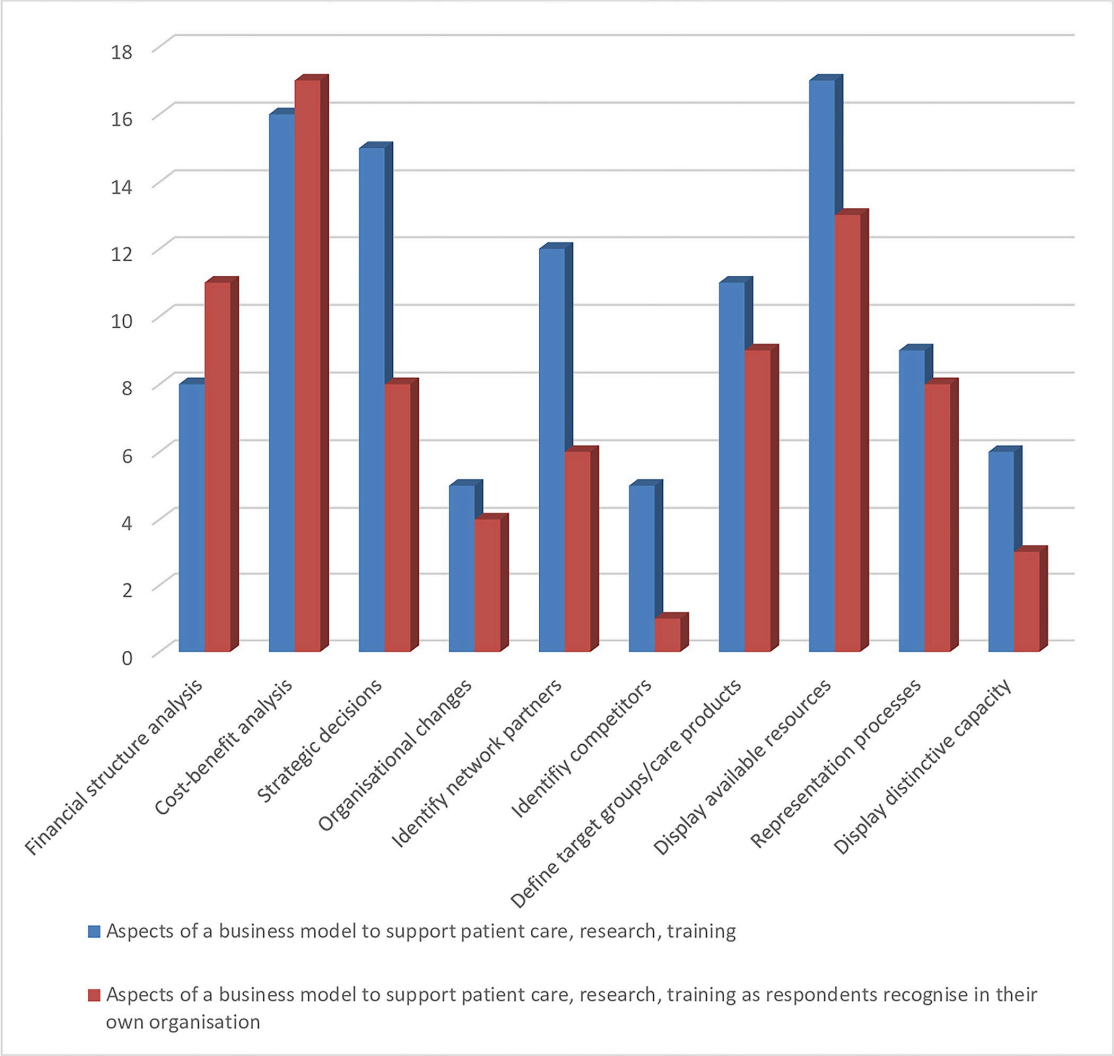
Supporting aspects of a business model. Aspects of a business model that support patient care, research and education.

The questionnaire consisted of two parts each with six questions. The questions were the same in both parts, but had to be answered in a different context. The first context concerned the use of a business model in a general sense. The second context concerned the actual use of a business model in the respondent’s daily practice. Pre-structured options were offered for 10 questions. The remaining two questions could be answered on a 5-point Likert scale (strongly agree—strongly disagree). To avoid the bias inherent in this design, each question offered an alternative answer or a brief explanation. The data from the questionnaires were uploaded into an Excel file. A senior researcher from IQ Healthcare at Radboud University Medical Centre in Nijmegen performed statistical analysis using IBM SPSS Statistics for Windows, version 25. This analysis produced frequency tables consisting of four columns: 1) absolute frequency, 2) relative frequency 3) validity percentage 4) cumulative percentage. The absolute frequency describes the number of times a particular value for a data item was observed. The second column expresses as a percentage how often a particular value for a variable (data item) was observed in relation to the total number of values for that variable. The relative frequency is calculated by dividing the absolute frequency by the total number of values for the variable. The data in the third and fourth columns were used for verification purposes only. The third column indicates which data items are valid and therefore useful for analysis. The fourth column adds up the percentages. The data from the first tables were presented graphically in bar charts (see Figs 1 and 2). The analysis focused on the perceived difference between the applicability of a business model in a general sense and its actual application in the respondents’ organisations. In other words, question 1 from part I was contrasted with question 8 from part II, question 2 with question 9, and so on. Differences were expressed in both numbers of responses and percentages. The interpretation of the results was carried out by two authors (Ester Cardinaal and Joey Truijens) and verified by two other authors (Patrick Jeurissen and Bart Berden).

## 4. Results

Respondents recognise that a business model can support the complex management of an AMC, but the design and use of business models varies. In general, respondents see more potential in the use of a business model than they experience in day-to-day practice. The majority consider a business model to be suitable for bringing about change, but see it used only sparingly used in their own AMC.

### 4.1 Use of a business model

Differences between the general perception of a business model and daily practice can be found in the areas of network partners, competitors, target groups/care products, processes and distinctive capacity. Respondents are less likely to see these components in their own practice, whereas they believe that a business model can make a positive contribution to these issues. 18 Respondents (75%) indicate that a business model can be used to make strategic decisions. Respondents also indicate that they see a role for business models in value creation and strategic workforce planning. See Fig 1.

### 4.2 Multiple business models in an AMC

22 respondents (91,7%) believe that it is possible to use multiple business models simultaneously within an organisation; 20 respondents (83.3%) see this in their own AMC. Multiple business models are interpreted in different ways: Six respondents (25%) say that the business model of the hospital is different from that of a department. Business models may also differ within departments, for example, to a greater of lesser extent externally focused. Only four respondents (17%) mention the use of multiple business models in relation to the different missions (patient care, research, education). Three respondents (12.5%) are critical of the use of multiple business models within an organisation. Reasons for this criticism vary from not desirable to not in the hospital’s interest or not in the department’s interest. Two respondents (8.3%) link different care models (acute, diagnostic, treatment, chronic) to the concept of a business model.

### 4.3 Supporting aspects of business models

Availability of resource (17 respondents, 70.8%), cost-benefit analysis (16 respondents, 66.7%), strategic decisions (15 respondents, 62.5%) and identification of network partners (12 respondents, 50%) are the most frequently mentioned elements of a business model that could support patient care, education, training and research.

However, respondents see the use of a business model mainly to support cost-benefit analysis (17 respondents, 70.8%) and the financial structure analysis (11 respondents, 45.8%). For the latter, they say that it is important for transparency of financial flows, but less so for operational excellence. Cost-benefit analysis is seen by most as a tool for making decisions in the context of actual operations. The opposite is felt for elements of strategic decision making, identification of network partners and available resources. 12 respondents consider the identification of network partners (50%) to be important in achieving patient-centred and integrated care. They consider strategic decision-making (15 respondents, 62.5%) necessary to set priorities and provide direction and guidance. They see the mapping of available resources (17 respondents, 70.8%) as necessary to manage and optimise operations through capacity planning. However, they see these elements less reflected in business models in their daily practice. See Fig 2.

### 4.4 A business model as a tool for change

23 respondents (95.8%) consider a business model an appropriate tool for change, but only 15 respondents (62.5%) see this reflected in their daily practice. Eight respondents (33.3%) indicate that business models are used to a very limited extent. A business model is mainly used for measuring key performance indicators, portfolio selection and cost-benefit analysis.

### 4.5 Using a business model to address current challenges

Most respondents indicated that they would use a business model to address challenges related to the ageing of the patient population, the emergence of medical technology, the shifting boundaries between primary, secondary and tertiary care, the emergence of preventive care, rising health care costs, research funding, tensions over resource allocation between core functions and labour market issues. Some respondents cautioned against using a business model to solve too many challenges at once.

## 5. Discussion

### 5.1 Summary of findings

Participants in our research recognised that a business model can support the complex management of an AMC. However, our research also shows that the design, use and understanding of business models varies from respondent to respondent. In addition, our respondents generally attribute more capabilities to the use of a business model than they experience in day-to-day practice. Specifically, the majority of respondents believe that a business model is capable of bringing about change, but see it used sparingly in their own AMCs. In conclusion, our research shows that business models are often perceived as too abstract in Dutch AMCs and are mainly used as a tool, especially for cost-benefit analysis, rather than as a means to bring about change to meet current internal and external challenges.

### 5.2 The context

The Dutch hospital landscape is characterised by a certain degree of stratification. AMCs are the largest hospitals with a leading position in tertiary highly complex patient care, biomedical scientific research, knowledge development and innovation. There are also large top clinical hospitals, which provide specialised care in addition to primary care and often have partnerships with other hospitals, including in the areas of physician training and scientific research. General hospitals are relatively small hospitals with a regional function that mainly provide primary care [16]. Finally, the Dutch hospital landscape includes smaller hospital organisations, including outpatient clinics, categorical hospitals that focus on a specific population group or disease and independent clinics for private, specialised medical care [17].

According to the Minister of Health, AMCs are unique in the Dutch health care system because they have been assigned public functions by law for which they receive specific funding [18]. This specific funding comes with both rights and obligations. Since the establishment of AMCs, politicians and other stakeholders have continued to debate this exceptional status of AMCs and their efficiency and transparency. In addition to these national pressures on the Dutch AMCs, they also have to deal with the global changes in supply and demand for health care. University hospitals are finding it increasingly difficult to maintain their current operations. An international comparison of 11 European AMCs shows that AMCs face significant challenges such as disruptive external pressures and ongoing financial conflicts between their patient care, research and teaching missions [2].

The literature increasingly points to the use of innovative business models to address these challenges [19, 20]. This makes the use of a business model potentially an important pillar for the management of AMCs. This was recently confirmed by IJntema et al. [21]. Their study shows that organisations achieve better performance in a changing environment by using a business model.

AMCs are large hospital organisations with complex structures due to the intertwining of missions, services and public functions [1, 5, 9]. Or as Peter Drucker once said: “*Even small healthcare institutions are complex*, *almost unmanageable places*… *Large health care institutions may be the most complex organisations in human history”* [1]. To successfully manage this complexity, these organisations are forced to use multiple business models simultaneously [5, 8, 22]. However, current AMC business models are based on a 19th century model [23]. At present, a variety of internal and external circumstances, challenges and objectives are forcing AMCs to rethink and adapt their operations [6, 24, 25]. In this context, Johansen et. al state that a fundamental change or transition is needed [26]. Hwang and Christensen urge the healthcare sector to think about business model innovation in order to reap the benefits of disruptive innovation [27]. However, there are few examples of disruptive process innovation in healthcare [26, 28], and the positive effects of such innovation often do not materialise in hospitals [29–31].

### 5.3 Barriers and challenges

From the results of our study we can derive some explanations as to why there are so few examples of disruptive innovation and disruptive business models in Dutch AMCs.

First, it could be due to a lack of in-depth knowledge about the adoption and implementation of business models. In a recent study by Kok et al. on attributes that contribute to the learning and improvement capacity of healthcare organisations, they note that what they call hardware elements (such as capacity management, resources and infrastructure) can facilitate change, but not initiate it [32]. Change also requires what they call software elements (such as psychological and social processes). It is conceivable that a business model alone (hardware) will not initiate change without sufficient attention to the organisation’s readiness to change (software). Along the same lines, the research of van den Hoed et al. adds four factors that contribute to change readiness in healthcare organisations 1) strategic direction 2) climate 3) leadership and 4) commitment to innovation [33]. It is plausible that the absence of these four factors hinders successful business model adoption. Secondly, the results of our study show that business models are not always understood in the same way and that business models are not always used in the same way and for the same purposes. This obviously complicates a collaborative approach to change in the care chain or within a single AMC where multiple business models are being implemented simultaneously [9]. In the IJntema study mentioned above, Dutch managers experience and emphasise the importance of using the same business model in the care chain to achieve and maintain better performance. At the same time, however, they note that this still varies widely in practice [21]. Finally, a European comparison of AMC governance has shown that AMCs struggle to adapt to changing circumstances [2]. A Dutch study found that this is (partly) due to the fact that working with (in) these large organisations is severely hampered by organisational complexity, lack of mutual trust and common interests, and perverse systemic incentives [34]. As noted in the study by van den Hoed et al. there are preconditions for the successful implementation of innovative business models [33].

Although the majority of business managers believe that the use of a business model can contribute to solving their current challenges and agree that it can be used as a tool to initiate change, the above obstacles can be seen as serious barriers to the successful implementation of disruptive or innovative business models.

### 5.4 Strengths and limitations

To the best of our best knowledge, this is the first study that provides exploratory insights into the use of business models in AMCs. All Dutch AMCs were represented, but in some cases only one person responded. Given the limited sample size, the generalisability of the results must be carefully considered [35]. In this study, sample size and data saturation are considered from the perspective that they should be operationalised in a way that is consistent with the exploratory research question [36]. This research is an exploratory study of the use of business models in AMCs. The aim is to use the exploratory findings to conduct more robust research. Future research on this topic could include a larger and more diverse sample of participants.

### 5.5 Implications

This research has provided a first insight into the use of business models in Dutch AMCs. Our research shows that the use of business models in the healthcare sector in general and in AMCs in particular is topical, but that the topic has not yet been fully explored. We can assume that there is room for improvement in terms of optimising the change potential of business models in AMCs worldwide. Before implementing an innovative business model, it is advisable for health care practitioners, policy makers or researchers to explicitly identify the environment in which the model will operate. In particular, it is important to ensure that the model is unambiguously interpreted. Work with collaborators to establish a clear starting point and definition. Then map readiness for change at all levels of the organisation (strategy, leadership, safety, commitment). If these factors are addressed, there may be fertile ground for the successful adoption of an innovative business model.

## Supporting information

S1 File Respondents per university medical centre. (DOCX)

S1 Questionnaire Questionnaire overview (translated from Dutch). (DOCX)

S1 Data (XLSX)
