# Supplementary material for: Use of business model potential in Dutch academic medical centres—A case study
Source: PLoS One. 2024 Mar 15;19(3):e0297966. doi: 10.1371/journal.pone.0297966 (PMC10942033; doi:10.1371/journal.pone.0297966)
Supplement: S1 File — (DOCX) [file pone.0297966.s001.docx]

**SUPPLEMENTARY FILE 1 Respondents per university medical centre**

| **University Medical Centre** | **Nr of respondents** |
| --- | --- |
| Amsterdam Universitair Medisch Centrum, Amsterdam | 2 |
| Erasmus Medisch Centrum, Rotterdam | 2 |
| Universitair Medisch Centrum, Groningen | 4 |
| Radboud Universitair Medisch Centrum, Nijmegen | 6 |
| Leids Universitair Medisch Centrum, Leiden | 1 |
| Maastricht Universitair Medisch Centrum | 4 |
| Universitair Medisch Centrum, Utrecht | 5 |
