# Supplementary material for: Use of business model potential in Dutch academic medical centres—A case study
Source: PLoS One. 2024 Mar 15;19(3):e0297966. doi: 10.1371/journal.pone.0297966 (PMC10942033; doi:10.1371/journal.pone.0297966)
Supplement: S1 Questionnaire — (DOCX) [file pone.0297966.s002.docx]

**SUPPLEMENTARY FILE 2 Questionnaire Overview** (translated from Dutch)

**PART I General view on (the use of) business models in a hospital**

Q1

If you were to deploy a business model in a hospital, what would you think of? Multiple answers possible.

- Analysis of the financial structure (e.g. origin, distribution, control)
- Cost/benefit analysis
- Strategic decisions
- Organisational changes
- Inclusion of network partners
- Inclusion of competitors
- Defining target groups (of care products)
- Recording of available resources
- Inclusion of processes within the organisation (e.g. training, planning decision processes)
- Display distinguishing capacity
- Other ....

Q2

Do you think that several business models could exist within one organisation? Please explain.

- Yes
- No

Q3

Could one or more of the following aspects support the core tasks (patient care, research, education) in a hospital? Please explain.

- Analysis of the financial structure (e.g. origin, distribution, control)
- Cost/benefit analysis
- Strategic decisions
- Organisational changes
- Inclusion of network partners
- Inclusion of competitors
- Defining target groups (of care products)
- Recording of available resources
- Inclusion of processes within the organisation (e.g. training, planning decision processes)
- Display distinguishing capacity
- Other ....

Q4

Could a business model be used as a steering tool to bring about change? Please explain.

- Yes
- No

Q5

To what extent could a business model contribute to the solution of the following challenges? Please use the scale below to indicate the extent to which you agree with the statement that a business model can contribute to solutions for the following challenges.

Strongly Strongly

Disagree Disagree Neutral Agree Agree N/A

Ageing
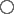

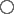

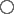

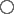

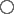

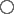


Emergence of medical technology

Blurring of boundaries between 1st, 2nd and 3rd line health care

Prevention

Rising healthcare costs

Research funding

Tension between core tasks and allocation of resources

Other

Q6

Which officials within a hospital could be concerned with setting up the business model(s)? Multiple answers possible.

- Healthcare professional
- Management
- Support staff (HR, Finance etc.)
- Manageing board
- Board
- Other

**PART II Using a business model in your organisation**

#### Q7

What is a business model used for in your organisation? Multiple answers possible.

- Analysis of the financial structure (e.g. origin, distribution, control)
- Cost/benefit analysis
- Strategic decisions
- Organisational changes
- Inclusion of network partners
- Inclusion of competitors
- Defining target groups (of care products)
- Recording of available resources
- Inclusion of processes within the organisation (e.g. training, planning decision processes)
- Display distinguishing capacity
- Other ....

Q8

Are there multiple business models within your organisation? Please explain.

- Yes
- No

Q9

Which of the following aspects support the core tasks (patient care, research, training) in your organisation? Please explain.

- Analysis of the financial structure (e.g. origin, distribution, control)
- Cost/benefit analysis
- Strategic decisions
- Organisational changes
- Inclusion of network partners
- Inclusion of competitors
- Defining target groups (of care products)
- Recording of available resources
- Inclusion of processes within the organisation (e.g. training, planning decision processes)
- Display distinguishing capacity
- Other ....

Q10

Is a business model used in your organisation as a management tool to bring about change? several Please explain.

- Yes
- No

Q11

To what extent does the (use of) a business model in your organisation contribute to solving the following challenges? Use the scale below to indicate to what extent you agree with the statement that a business model can contribute to solutions for the following challenges.

Strongly Strongly

Disagree Disagree Neutral Agree Agree N/A

Ageing
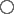

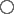

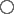

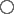

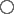

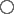


Emergence of medical technology

Blurring of boundaries between 1st, 2nd and 3rd line health care

Prevention

Rising healthcare costs

Research funding

Tension between core tasks and allocation of resources

Other

Q12

Which officials within your organisation are involved in setting up the business model(s)? Please explain.

- Healthcare professional
- Management
- Support staff (HR, Finance etc.)
- Manageing board
- Board
- Other

**BACKGROUND INFORMATION**

The changing healthcare landscape brings various challenges for hospitals. Solutions are being sought at various levels, including the organisational level. There are indications in the scientific literature that the use of a business model can contribute to the success of an organisation/hospital. An example of a hospital that successfully applies a business model is the Aravind Eye Hospital in India, which specialises in performing cataract operations and is able to offer these at no cost to a proportion of its patients because the paying proportion of patients generates enough cash flow (Govindarajan, V., & Ramamurti, R. (2013). Delivering world-class health care, affordably. Harvard Business Review, 117–122).

A business model maps out various business aspects and can help with management. The model can include organisational, financial, operational but also idealistic components, even though no unambiguous definition emerges from the scientific literature (Wirtz, B. W., Pistoia, A., Ullrich, S., & Göttel, V. (2016). Business Models: Origin, Development and Future Research Perspectives. Long Range Planning, 49(1), 36–54. <https://doi.org/10.1016/j.lrp.2015.04.001>).

In 2020, the book "Understanding Hospitals in Changing Health Systems" was published. A "model of care" is a theoretical construct that describes how healthcare is delivered. A model of care is about the organisation of care and takes various forms depending on the deployment of production factors (infrastructure, capital, equipment, personnel, facilities, etc.). A model of care can, for instance, deal with the patient's path through the hospital's services, or with clinical decision rules (which treatments are given to which patients, at which time, and by whom). However, it is not possible to make decisions solely on the basis of a model of care, especially medium to long-term decisions. Of course, when choosing a desired model of care, it is important to think about its costs and benefits. This means not only looking at how the care is organised, but also at its economic and financial value. And then we talk about a business model. It goes without saying that a business model in a hospital is different from a business model in a commercial setting. It is important to see a hospital business model in conjunction with the model of care mentioned by Duran et al. A model of care will always have to be placed within a business model in order to make its financial value clear. Conversely, a hospital's business model must always include a model of care. By distinguishing between the two concepts (models of care and business models), it becomes possible for policymakers to separate changes in the care process from economic interests (Durán, A., & Wright, S. (Eds.). (2020). Understanding Hospitals in Changing Health Systems. doi:10.1007/978-3-030-28172-4).
